# Isolation and Characterization of Carbapenem-Resistant *Escherichia coli* Carrying *bla*_NDM_ and *mcr-1* from Recurrent Urinary Tract Infection Patient

**DOI:** 10.1155/2023/6640009

**Published:** 2023-08-30

**Authors:** Liang Chen, Jiyong Jian, Zeqiang Xie, Ping Zhao, Man Zhang

**Affiliations:** ^1^Clinical Laboratory Medicine, Beijing Shijitan Hospital, Capital Medical University, Beijing, China; ^2^Peking University Ninth School of Clinical Medicine, Beijing, China; ^3^Beijing Key Laboratory of Urinary Cellular Molecular Diagnostics, Beijing, China

## Abstract

**Objective:**

The emergence of carbapenem-resistant *E. coli* (CRECO), leading to few antibacterial drugs available for CRECO infection. In this study, we report three carbapenem-resistant *Escherichia coli* (*E. coli*) isolates coproducing *bla*_NDM_ and *mcr-1* from patients with recurrent urinary tract infection (RUTI). Carbapenem-resistant *E. coli* strains, E55, E84, and E85, were isolated from the urine sample of RUTI patients.

**Methods:**

Antimicrobial susceptibility testing (AST) was conducted with VITEK-2 compact system and Kirby–Bauer (K-B) disk diffusion method. The ESBL test was detected by the disk diffusion method. The EDTA-modified carbapenem inactivation method (eCIM) and modified carbapenem inactivation method (mCIM) were performed for screening the carbapenemase. Multilocus sequence typing (MLST) was performed for molecular typing of the strains. The resistance genes were detected by PCR.

**Results:**

The three isolates were all susceptible to tigecycline and nitrofurantoin. The *bla*_NDM-1_, *bla*_CMY-6_, *bla*_TEM-1_ and *bla*_CTX-M-1_, *mcr-1*, and porin loss expression of outer membrane protein F (*OmpF*) were detected in E55, which was assigned to ST2. The E84 and E85 were identified as ST471 carrying *bla*_NDM-5_, *bla*_CTX-M55_, and *bla*_TEM-1_ and the quinsolone-resistant genes *aac(6′)-Ib-cr* and *mcr-1*.

**Conclusion:**

To our knowledge, our study is the first to report carbapenem-resistant *E. coli* strains carrying *bla*_NDM_ and *mcr-1* from urine of the recurrent urinary tract infection patients. These *E. coli* strains carrying *bla*_NDM_ and *mcr-1* should be closely monitored.

## 1. Introduction

*Escherichia coli* (*E. coli*) is an important pathogen in the clinical infectious disease, especially in the urinary tract infections (UTIs) [1]. The emergence of carbapenem-resistant *E. coli* (CRECO) leads to few therapeutic options available. Carbapenem antibiotics play an important role in treatment of these multiple drug-resistant (MDR) bacterial infections and have been widely used in clinical practices [2]. Carbapenem-resistant *Enterobacteriaceae* (CRE) remains a global public health threat, like tuberculosis (TB) [3]. New Delhi metallo-*β*-lactamase (NDM) is an important carbapenemase that confers resistance to almost all *β*-lactams. Polymyxins are considered among the last therapeutic options to therapy the serious infections caused by CRECO [4]. However, the first report about mobile colistin resistance 1 (*mcr-1*) was published in 2016, detected from ECO and *Klebsiella pneumoniae* isolates recovered from animals and patients in China, which is responsible for colistin resistance [5]. Since then, more and more literatures were published to report the detection of the *mcr-1* gene from more sites from animals and humans in Europe, Canada, Vietnam, Hong Kong, and Taiwan [6–10]. But there are few reports of *mcr*_s_ detected from clinical patient samples, especially the urine of patients with recurrent urinary tract infection (RUTI). Here, we report three isolated *E. coli* strains, which coproduce *mcr-1* and *bla*_NDMs_ from patients with RUTI.

## 2. Materials and Methods

### 2.1. Bacterial Isolation and Identification

Three *E. coli* strains, E55, E84, and E85, were isolated from the urine sample of RUTI patients. The three isolates were identified by the VITEK-2 compact system (bioMérieux, France) and 16S rRNA sequencing.

### 2.2. Antimicrobial Susceptibility Testing

In vitro antimicrobial susceptibility testing (AST) was conducted using VITEK-2 compact system (bioMérieux, France) and Kirby–Bauer (K-B) disk diffusion method (filter paper from Oxoid), using *E. coli* (ATCC 25922) as the control. The results were interpreted following the Clinical and Laboratory Standards Institute (CLSI), and colistin and tigecycline were interpreted according to the European Committee on Antimicrobial Susceptibility Testing (EUCAST) (https://www.eucast.org/).

### 2.3. Phenotypic Detection of ESBL and Carbapenemase

Recommended by the CLSI [11], ESBL production was confirmed by combined disk approach, and phenotypic screening for preliminarily determining whether the strains produced metallo-carbapenemase was performed in accordance with the modified carbapenem inactivation method (mCIM) and EDTA-modified carbapenem inactivation method (eCIM).

### 2.4. DNA Extraction

Colonies of the clinical *E. coli* strains were transferred to a microcentrifuge tube with sterile distilled water. The samples were boiled to prepare the DNA templates for polymerase chain reaction (PCR).

### 2.5. Antimicrobial Resistance Gene Identification

The presence of the acquired resistance genes, including Ambler A (*bla*_NMC_, *bla*_IMI_, *bla*_SME_, *bla*_KPC_, and *bla*_GES_), Ambler B (*bla*_IMP_, *bla*_VIM_, *bla*_NDM_, *bla*_SIM_, *bla*_SPM_, and *bla*_GIM_), Ambler D (*bla*_OXA48_), AmpC (*bla*_CMY_, *bla*_DHA_, *bla*_ACC_, *bla*_EBC_, *bla*_FOX_, and *bla*_MOX_), ESBL (*bla*_TEM_, *bla*_CTX_, and *bla*_SHV_), quinolone resistance gene (*aac(6′)-Ib-cr*, *qnrA*, *qnrB*, *qnrC*, *qnrD*, and *qnrS*), aminoglycoside resistance genes (*aac(6′)-Ib*, *armA*, and *rmtB*), colistin resistance genes (*mcr1-5*), and the loss of outer membrane prion gene (*OmpA*, *OmpC*, and *OmpF*) was screened by PCR using primers (supplementary table (available here)), and then the positive products were validated with Sanger sequencing.

### 2.6. Molecular Typing

Multilocus sequence typing (MLST) was performed using eight housekeeping genes (*din B, icdA, pabB, polB, putP, trpA, trpB,* and *uidA*) of *E. coli* which were amplified using primers showed in online databases (https://www.Pasteur.fr/mlst.). PCR products were sequenced, and STs are available using online database tools.

## 3. Results

### 3.1. Bacterial Identification

The three strains E55, E84, and E85 were all *E. coli*, identified by VITEK 2 compact and confirmed by 16S rRNA sequencing.

### 3.2. Antimicrobial Susceptibility Testing

The three strains showed multidrug-resistant phenotypes. E55 was resistant to most of the antibiotics, excluding tigecycline and nitrofurantoin. E84 was only susceptible to amikacin, doxycycline, tigecycline, trimethoprim-sulfamethoxazole, gentamicin, minocycline, and nitrofurantoin. E85 was only susceptible to trimethoprim-sulfamethoxazole, gentamicin, nitrofurantoin, tigecycline, and amikacin. The results of the antibiotics tested are presented in Table 1.

### 3.3. Characterization of Carbapenem-Resistant *E. coli*

ESBL tests of the three strains were all negative. mCIM tests of the three strains were all positive, then eCIM tests were done, and the results were all positive too (Table 2).

To determine the mechanism of colistin resistance and carbapenem resistance, we initially investigated the presence of resistance genes. We identified that there were the beta-lactam resistance genes *bla*_NDM-1_, *bla*_CMY-6_, *bla*_TEM-1_ and *bla*_CTX-M-1_, and *mcr-1*, accompanied by the loss of *OmpF* in E55, and the MLST typing of E55 belongs to ST2. E84 and E85 were identified as ST471 carrying *bla*_NDM-5_, *bla*_CTX-M55_, and *bla*_TEM-1_, *aac(6′)-Ib-cr,* and *mcr-1*. The results of antimicrobial resistance genes and MLST are presented in Table 2.

## 4. Discussion

UTI is an inflammation of the urinary tract caused by bacteria. It is one of the most common bacterial infection diseases [1]. In a survey of 289 female patients with recurrent urinary tract infections from 2006 to 2014, it was found that 71% of persistent and 47% of recurrent urinary tract infections were caused by *E. coli* [12]. Gordon and Jones reported that the isolation rate of *E. coli* in UTI was as high as 47% in North America, Europe, and Latin America from a retrospective investigation of SENTRY program in 2000 [13]. Some other studies had shown that among the pathogenic bacteria of urinary tract infection detected from 2016 to 2017, *E. coli* ranked no. 1, accounting for 28.85%. The data reported by other domestic literatures were basically consistent [14, 15]. In this study, we found three carbapenem-resistant *E. coli* isolates coproducing *bla*_NDM_ and *mcr-1* from patients with recurrent urinary tract infection (RUTI).

The three strains were all MDR isolates. Compared with E84 and E85, E55 showed more resistance to antibiotics; it was only susceptible to tigecycline and nitrofurantoin. While nitrofurantoin showed susceptibility to the three strains, it suggested that nitrofurantoin might be a treatment option for treatment of RUTI caused by colistin and carbapenem-resistant *E. coli*.

In our study, the three *E. coli* isolates showed multidrug resistance not only because of *bla*_NDM_ and *mcr-1* gene but also due to other resistance mechanisms, for example, the *bla*_CMY-6_, *bla*_TEM-1_, and *bla*_CTX-M-1_, which code the AmpC and ESBLs in E55; the *bla*_CTX-M55_ and *bla*_TEM-1_ and *aac(6′)-Ib-cr+* which code ESBLs and the quinolone resistance genes in E84 and E85. But in E55, quinolone-resistant genes and aminoglycoside-resistant genes were not detected, and the isolate showed resistance to the two antibiotics, maybe because of the deletion of the *OmpF* gene. Most of the membrane porins that *β*-lactam antibiotics can pass through are mainly OmpF and OmpC, which are characterized by a significant reduction in the permeability of negatively charged substances, while a positive charge can promote solute passage. It is suggested that the loss of porin is related to the resistance of bacteria to *β*-lactam antibiotics. It has been found that the loss of pore protein contributes more to carbapenem resistance than ESBLs or carbapenemases' presence. Because the drugs targeting porin loss are limited, related drugs targeting the mechanism must be developed to address the carbapenem resistance issue of pathogenic bacteria [16].

In addition, our results showed that E55 coproduced *mcr-1* and *bla*_NDM1_, while E84 and E85 coproduced *mcr-1* and *bla*_NDM5_. The bacteria carried the New Delhi metallo-*β*-lactamase gene (*bla*_NDM−1_) which was first identified in 2009 [17], and then *E. coli* carried *mcr-1* which was first identified in 2016 [5]. Because the *mcr-1* and *bla*_NDM_ genes can disseminate widely around the world and across species [5], *E. coli* with *mcr-1* or *bla*_NDM_ can also be detected gradually from some patients, but the related reports are rare. MCR-1 and NDM-5 coproducing isolate was first reported from a duck sample [18], but soon reported from urine [19–22], blood [21], ascites [21], and abdominal drainage [22]. And soon more literatures had reported the coexistence of *mcr-1* and *bla*_NDM-5_ genes in clinic origin and animal origin. Han et al. represented the first report of a wild-derived *E. coli* strain harboring *mcr-1* and *bla*_NDM-5_ genes simultaneously [23]. So far, strains coproducing NDM-1 and MCR-1 had been reported from blood and feces [24–26]. However, there is no report from urine. As we know, this is the first report of carbapenem-resistant *E. coli* strain carrying bla_NDMs_ and mcr*-1* from urine of the RUTI patients.

In this study, the ESBL tests of the three carbapenem-resistant *E. coli* strains were negative, but *bla*_TEM-1_ and *bla*_CTX-M-1_ were detected in E55, *bla*_TEM-1_ and *bla*_CTX-M-55_ which were detected in E84 and E85. The reason is the production of carbapenemase by bacteria which cannot be inhibited by any of the *β*-lactamase inhibitors. So, under the action of carbapenemase and other resistance mechanisms, the antibiotics with enzyme inhibitors, for example, ticarcillin/clavulanic acid, piperacillin-tazobactam, ampicillin/sulbactam, and cefperazone-sulbactam, also showed resistance.

The results of mCIM and eCIM are consistent with gene detection in the study. The specificity and sensitivity of mCIM and eCIM can reach 100%. It is simple, cost-effective, criteria clear and can be available easily in any laboratory. The mCIM and eCIM were recommended by CLSI in 2017. The mCIM and eCIM have become a useful tool in microbiology laboratories, but its limitation is time-consuming [27].

So far, ST2373, ST131, ST744, and ST19 were reported in *E. coli* carrying *bla*_NDM-1_ and *mcr-1* by MLST typing, and ST405, ST167, ST156, ST25, and ST206 were reported in *E. coli* carrying *bla*_NDM-5_ and *mcr-1* by MLST typing [18–20]. In this study, the MLST typing of E55 belongs to ST2, while E84 and E85 belong to ST471, different from the abovementioned types. These isolates exhibited the clonal diversity, and the prevalence of these isolates was not caused by clonal dissemination.

To this study, there were some limitations. First, in our study, we detected *ompA*, *ompC*, and *ompF* genes deletion but did not determine the expression level of *omps* using RT-PCR or western blot. Second, pulsed field gel electrophoresis analysis of S1 nuclease-digested DNA (S1-PFGE), followed by southern blotting should be conducted to identify the location of *mcr-1* and *bla*_NDMs_-carrying plasmids. Finally, the genetic characteristics of the *mcr-1*- and *bla*_NDMs_-harboring plasmids also should be analyzed.

## 5. Conclusion

As we know, this is the first report of carbapenem-resistant *E. coli* strain carrying bla_NDMs_ and mcr*-1* from urine of the RUTI patients. The coexistence of *mcr*-1 and carbapenemase genes in *E. coli* may weaken the effectiveness of therapy and pose a potential threat to public health. Constant surveillance of polymyxin- and carbapenem-resistant organisms is imperative in order to prevent the spread of mobile antibiotic resistance mechanisms.

## Figures and Tables

**Table 1:** Resistance profiles of different isolates of carbapenem-resistant *Escherichia coli*.

Antibiotics	E55			E84			E85		
	MIC (mg/L)	K-B (mm)	Interpretive categories	MIC (mg/L)	K-B (mm)	Interpretive categories	MIC (mg/L)	K-B (mm)	Interpretive categories
Tikacillin clavulanic	≥128	—	R	≥128	—	R	≥128	—	R
Piperacillin-tazobactam	≥128	—	R	≥128	—	R	≥128	—	R
Ceftazidime	≥64	—	R	≥64	—	R	≥64	—	R
Cefperazone-sulbactam	≥64	—	R	≥64	—	R	≥64	—	R
Cefepime	16	—	R	≥32	—	R	≥32	—	R
Aztreonam	16	—	R	≥64	—	R	≥64	—	R
Imipenem	≥16	—	R	≥16	—	R	≥16	—	R
Meropenem	8	—	R	≥16	—	R	≥16	—	R
Ertapenem	≥8	—	R	≥8	—	R	≥8	—	R
Amikacin	≥64	—	R	16	—	S	16	—	S
Tobramycin	≥16	—	R	≥16	—	R	≥16	—	R
Ciprofloxacin	≥4	—	R	≥4	—	R	≥4	—	R
Levofloxacin	≥8	—	R	≥8	—	R	≥8	—	R
Doxycycline	≥16	—	R	2	—	S	≥16	—	R
Minocycline	≥8	—	I	≤1	—	S	≥16	—	R
Tigecycline	≤0.5	—	S	≤0.5	—	S	≤0.5	—	S
Colistin	≥8	—	R	4	—	R	4	—	R
Trimethoprim-sulfamethoxazole	≥320	—	R	≤20	—	S	≤20	—	S
Ampicillin	≥32	—	R	≥32	—	R	≥32	—	R
Ampicillin/sulbactam	≥32	—	R	≥32	—	R	≥32	—	R
Cefazolin	≥64	—	R	≥64	—	R	≥64	—	R
Cefotetan	≥64	—	R	≥64	—	R	≥64	—	R
Ceftriaxone	≥64	—	R	≥64	—	R	≥64	—	R
Gentamycin	≥32	—	R	≤1	—	S	≤1	—	S
Nitrofurantoin	32	—	S	≤16	—	S	≤16	—	S
Doripenem	—	10	R	—	10	R	—	6	R
Fosfomycin	—	6	R	—	6	R	—	6	R
Mecillinam	—	11	R	—	13	I	—	10	R

**Table 2:** Characterization of carbapenem-resistant *Escherichia coli* carrying *bla*_NDM_ and *mcr-1*.

	E55	E84	E85
MLST	ST2	ST471	ST471
Resistance gene	*bla*_NDM-1_, *bla*_CMY-6_, *bla*_TEM-1_, *bla*_CTX-M-1_, *mcr-1*, and *OmpF*	*bla*_NDM-5_, *bla*_TEM-1_, *bla*_CTX-M-55_, *aac(6′)-Ib-cr+*, and *mcr-1*	*bla*_NDM-5_, *bla*_TEM-1_, *bla*_CTX-M-55_, *aac(6′)-Ib-cr+*, and *mcr-1*
ESBLtest	Negative	Negative	Negative
mCIM	Positive	Positive	Positive
eCIM	Positive	Positive	Positive

## Data Availability

The data used to support the findings of this study are included within the article.

## Supplementary Materials

Supplementary Materials Supplementary materials are available at the Canadian Journal of Infectious Diseases and Medical Microbiology online. Supplementary table: the primers used to amplify the resistance genes.
