# Supplementary material for: Isolation and Characterization of Carbapenem-Resistant Escherichia coli Carrying blaNDM and mcr-1 from Recurrent Urinary Tract Infection Patient
Source: Can J Infect Dis Med Microbiol. 2023 Aug 30;2023:6640009. doi: 10.1155/2023/6640009 (PMC10482531; doi:10.1155/2023/6640009)
Supplement: Supplementary Materials — are available at the Canadian Journal of Infectious Diseases and Medical Microbiology online. Supplementary table: the primers used to amplify the resistance genes. [file 6640009.f1.docx]

**Supplementary Table. The primers used to amplify the resistance genes**

| Type | Gene | Sequence(5′-3′) | Length of ampliﬁcation fragment(bp) | Annealing temperature  （°C） |
| --- | --- | --- | --- | --- |
| Ambler A | *bla*_KPC_ | F: 5′-TGTCACTGTATCGCCGTC-3′  R: 5′-CTCAGTGCTCTACAGAAAACC -3′ | 1010 | 58 |
|  | *bla*_GES_ | F: 5′- GTTTTGCAATGTGCTCAACG-3′  R: 5′- TGCCATAGCAATAGGCGTAG-3′ | 371 | 54 |
|  | *bla*_NMC_ | F: 5′-GCATTGATATACCTTTAGCAGAGA-3′  R: 5′-CGGTGATAAAATCACACTGAGCATA-3′ | 2158 | 54 |
|  | *bla*_IMI_ | F: 5′- ATAGCCATCCTTGTTTAGCTC-3′  R: 5′- TCTGCGATTACTTTATCCTC-3′ | 818 | 54 |
|  | *bla*_SME_ | F: 5′-GCATTGATATACCTTTAGCAGAGA-3′  R: 5′-CGGTGATAAAATCACACTGAGCATA-3′ | 2158 | 54 |
| Ambler B | *bla*_NDM_ | F: 5′- TCGCCCCATATTTTTGCTACAG-3′  R: 5′- CGATCCTTCCAACTCGTCGC-3′ | 1012 | 54 |
|  | *bla*_IMP-1_ | F: 5′- TGAGCAAGTTATCTGTATTC-3′  R: 5′- TTAGTTGCTTGGTTTTGATG-3′ | 740 | 55 |
|  | *bla*_SIM-1_ | F: 5′- TACAAGGGATTCGGCATCG-3′  R: 5′- TAATGGCCTGTTCCCATGTG-3′ | 571 | 54 |
|  | *bla*_IMP-2_ | F: 5′- GGCAGTCGCCCTAAAACAAA-3′  R: 5′- TAGTTACTTGGCTGTGATGG-3′ | 737 | 55 |
|  | *bla*_VIM-1_ | F: 5′- TTATGGAGCAGCAACCGATGT-3′  R: 5′- CAAAAGTCCCGCTCCAACGA-3′ | 920 | 55 |
|  | *bla*_VIM-2_ | F: 5′-AAAGTTATGCCGCACTCACC-3′  R: 5′-TGCAACTTCATGTTATGCCG-3′ | 865 | 55 |
|  | *bla*_SPM_ | F: 5′- CCTACAATCTAACGGCGACC-3′  R: 5′′-TCGCCGTGTCCAGGTATAAC-3′ | 650 | 54 |
|  | *bla*_GIM_ | F: 5′-AGAACCTTGACCGAACGCAG-3′  R: 5′- ACTCATGACTCCTCACGAGG-3′ | 748 | 54 |
| Ambler D | b*la*_OXA48_ | F: 5′- TTGGTGGCATCGATTATCGG-3′  R: 5′- GAGCACTTCTTTTGTGATGGC-3′ | 438 | 55 |
| ESBL | *bla*_TEM_ | F: 5′- ATGAGTATTCAACATTTCCG-3′  R: 5′- CCAATGCTTAATCAGTGAGG-3′ | 859 | 55 |
|  | *bla*_CTX-M-1_ | F: 5′- ATGGTGACAAAGAGAGTGCA-3′  R: 5′-CCCTTCGGCGATGATTCTC-3′ | 863 | 55 |
|  | *bla*_CTX-M-2_ | F: 5′- GGTTTAAAAAATCACTGCGTC-3′  R: 5′-TTGGTGACGATTTTAGCCGC-3′ | 833 | 55 |
|  | *bla*_SHV_ | F: 5′-AGGATTGACTGCCTTTTTG-3′  R: 5′- ATTTGCTGATTTCGCTCG-3′ | 392 | 55 |
| AmpC | *bla*_CMY_ | F: 5′-TGG CCA GAA CTG ACA GGC AAA-3′  R: 5′- TTT CTC CTG AAC GTG GCT GGC-3′ | 462 | 64 |
|  | *bla*_DHA-1_ | F: 5′-AAC TTT CAC AGG TGT GCT GGG T-3′  R: 5′- CCG TAC GCA TAC TGG CTT TGC-3′ | 405 | 55 |
|  | *bla*_ACC_ | F: 5′- AAC AGC CTC AGC AGC CGG TTA-3′  R: 5′-TTC GCC GCAATC ATC CCT AGC-3′ | 346 | 55 |
|  | *bla*_EBC_ | F: 5′-TCG GTA AAG CCG ATG TTG CGG-3′  R: 5′-CTT CCA CTG CGG CTG CCA GTT-3′ | 302 | 55 |
|  | *bla*_FOX_ | F: 5′-AAC ATG GGG TAT CAG GGA GAT G-3′  R: 5′- CAA AGC GCG TAA CCG GAT TGG-3′ | 190 | 55 |
|  | *bla*_MOX_ | F: 5′- GCT GCT CAA GGA GCA CAG GAT-3′  R: 5′- CAC ATT GAC ATA GGT GTG GTG C-3′ | 520 | 55 |
| aminoglycoside | *aac(6' )-Ib* | F:5'-TATGAGTGGCTAAATCGAT-3'  R:5'-CCCGCTTTCTCGTAGCA -3' | 395 | 55 |
|  | *armA* | F:5'-CCGAAATGACAGTTCCTATC-3'  R:5'-GAAAATGAGTGCCTTGGAGG-3' | 846 | 55 |
|  | *rmtB* | F:5'-ATGAACATCAACGATGCCCT -3'  R:5'- CCTTCTGATTGGCTTATCCA-3' | 769 | 55 |
| quinolone | *aac(6' )-Ib-cr* | F:5'-ATATGCGGATCCAATGAGCAACGCAA AAACAAAGTTAG -3'  R:5'-ATAGCGAATTCTTAGGCATCACTGCGTGTTCGCTC-3' | 544 | 54 |
|  | *qnrS* | F:5'- GCAAGTTCATTGAACAGGGT -3'  R:5'- TCTAAACCGTCGAGTTCGGCG -3' | 428 | 58 |
|  | *qnrB* | F: 5'- GATCGTGAAAGCCAGAAAGG -3'  R: 5'- ACGATGCCTGGTAGTTGTCC -3' | 469 | 53 |
|  | *qnrA,* | F: 5'- ATTTCTCACGCCAGGATTTG -3'  R: 5'-GAGATTGGCATTGCTCCAGT-3' | 413 | 53 |
|  | *qnrC* | F:5'-GGGTTGTACATTTATTGAATC -3'  R:5'-TCCACTTTACGAGGTTCT -3' | 447 | 47 |
|  | *qnrD* | F:5'-CGAGATCAATTTACGGGGAATA -3'  R:5'- AACAAGCTGAAGCGCCTG -3' | 582 | 54 |
| the loss of OMP | Omp F | F: 5′-CGCCAAATCGATAAAACTT-3′  R: 5′-TAAGTCGAACATCCCCATG-3′ | 115 | 55 |
|  | Omp C | F: 5′-AAGGCAGCATCACCAAAGT-3′  R: 5′-TCAGTCGGCAAGTCCATTC-3′ | 137 | 55 |
|  | Omp A | F: 5′-GCCGCTCCGAAAGACAACA-3′  R: 5′-CCAAAAGCACCTGCACCCA-3′ | 126 | 55 |
| colistin | *mcr-1* | F: 5′-CGGTCAGTCCGTTTGTTC-3′  R: 5′-CTTGGTCGGTCTGTAGGG-3′ | 309 | 55 |
|  | *mcr-2* | F: 5′-TGTTGCTTGTGCCGATTGGA-3′  R: 5′-AGATGGTATTGTTGGTTGCTG-3′ | 567 | 65 |
|  | *mcr-3* | F:5′-TTGGCACTGTATTTTGCATTT-3′  R: 5′-TTAACGAAATTGGCTGGAACA-3′ | 542 | 50 |
|  | *mcr-4* | F: 5′-ATTGGGATAGTCGCCTTTTT-3′  R: 5′-TTACAGCCAGAATCATTATCA-3′ | 487 | 55 |
|  | *mcr-5* | F: 5′-ATGCGGTTGTCTGCATTTATC-3′  R:5′-TCATTGTGGTTGTCCTTTTCTG-3′ | 1644 | 50 |
